# An Approach to Reducing Information Loss and Achieving Diversity of Sensitive Attributes in k-anonymity Methods

**DOI:** 10.2196/ijmr.2140

**Published:** 2012-11-13

**Authors:** Sunyong Yoo, Moonshik Shin, Doheon Lee

**Affiliations:** 1 Department of Bio and Brain Engineering, KAISTDaejeonKorea, Republic Of

**Keywords:** k-anonymity, l-diversity, Information loss, Conditional entropy, Mutual information

## Abstract

Electronic Health Records (EHRs) enable the sharing of patients’ medical data. Since EHRs include patients’ private data, access by researchers is restricted. Therefore k-anonymity is necessary to keep patients’ private data safe without damaging useful medical information. However, k-anonymity cannot prevent sensitive attribute disclosure. An alternative, *l*-diversity, has been proposed as a solution to this problem and is defined as: each Q-block (ie, each set of rows corresponding to the same value for identifiers) contains at least *l* well-represented values for each sensitive attribute. While *l*-diversity protects against sensitive attribute disclosure, it is limited in that it focuses only on diversifying sensitive attributes.

The aim of the study is to develop a k-anonymity method that not only minimizes information loss but also achieves diversity of the sensitive attribute.

This paper proposes a new privacy protection method that uses conditional entropy and mutual information. This method considers both information loss as well as diversity of sensitive attributes. Conditional entropy can measure the information loss by generalization, and mutual information is used to achieve the diversity of sensitive attributes. This method can offer appropriate Q-blocks for generalization.

We used the adult database from the UCI Machine Learning Repository and found that the proposed method can greatly reduce information loss compared with a recent *l*-diversity study. It can also achieve the diversity of sensitive attributes by counting the number of Q-blocks that have leaks of diversity.

This study provides a privacy protection method that can improve data utility and protect against sensitive attribute disclosure. The method is viable and should be of interest for further privacy protection in EHR applications.

## Introduction

Society is experiencing exponential growth in the amount of health information. However, this information is distributed across multiple sites, held in a variety of paper and electronic formats, and represented as mixtures of narrative and structured data. Electronic Health Records (EHRs) have been introduced as a method for improving communication between health care providers and improving access to patient data. This use of EHRs has now enabled large and complicated databases of health records to be used for medical and other research. However, as medical record data become more accessible, protecting patient privacy is an increasing concern that should not be overlooked or understated [1-4].

For patient health information to be de-identified, the Health Insurance Portability and Accountability Act (HIPAA) in the United States suggests the “Safe Harbor” technique, which requires 18 data elements to be removed [5,6]. Doing this can protect the confidentiality and privacy of research subjects. De-identification methods have been proposed for removal of identifiers and in general are performed by the following two steps. First, personal identifiers are located within a database. Second, these identifiers are masked, coded, and/or replaced with irreversible values to unauthorized personnel. However, de-identification methods have tended to be quite faulty as the possibility remains of re-identifying a patient by linking or matching the data to other data or by looking at unique characteristics found in the released data.

Avoiding re-identification requires the use of an anonymization method that prevents the data from being linked for identification of the patient. One popular anonymization method is k-anonymity, proposed by Samarati and Sweeny. A dataset satisfies k-anonymity if each record is indistinguishable from at least k-1 other records with respect to certain identifying attributes. This process is usually performed by suppressing or generalizing database entries [7-10].

While k-anonymity protects against identity disclosure, it is not sufficient for preventing sensitive attribute disclosure. To solve this problem, *l*-diversity has been proposed [11,12]. This method requires that each Q-block has at least *l* well-represented values for each sensitive attribute. While *l*-diversity protects against sensitive attribute disclosure, it has a limitation in that it focuses only on diversifying sensitive attributes. However, generalizing attributes leads to an information loss, so reducing the amount of information loss is also important [8,13].

In this paper, we propose a practical method that reduces information loss but still achieves diversity of sensitive attributes. This method is based on conditional entropy and mutual information. Conditional entropy can measure the information loss by generalization between the original database and a generalized database, while mutual information between the generalized database and sensitive attributes can be used to achieve the necessary diversity of sensitive attributes. By applying this method, we were able to offer appropriate Q-blocks for generalization. We used the adult database from the UCI Machine Learning Repository to evaluate the proposed method.

### Related Work

Privacy has become an increasingly salient political issue and considerable progress has been made with de-identification. In general, de-identification methods aim to remove a patient’s personal information and many other types of PHI (Protected Health Information). The de-identification process means that only explicit identifiers are hidden or removed. Despite using various measures to de-identify health records, it is possible to re-identify them in a large number of cases by using computerized network databases containing voter registration records, hospital discharge records, commercially available databases, and other sources. Indeed, it is likely that between 63% (Golle 2006) and 87% (Sweeney 2000) of the population of the United States could be uniquely identified by using only gender, ZIP code, and date of birth [14,15].

This kind of attack is called a linking attack. We assumed that an individual has a de-identified database containing some clinical data and that those databases also contain attributes (birth, gender, and zip code). If we could get access to an identification database or construct one from public data sources with the same attributes as the database, then it would be easier to link two databases and re-identify the individuals in the research database [16]. This linkage is performed with a set of quasi-identifier (QI) attributes that are in both datasets. In Table 1, *work* and *country* are QI attributes.

**Table 1:** An example of an original data table.

Index	Quasi-identifier (QI)		Sensitive
	Work	Country	Disease
1	Private	USA	Heart Disease
2	State-gov	Mexico	Cancer
3	Local-gov	Brazil	Cancer
4	Federal-gov	USA	Flu
5	Private	Canada	Heart Disease
6	Self-emp-not-inc	Canada	Heart Disease
7	Self-emp-inc	USA	Flu
8	Private	USA	Heart Disease
9	State-gov	Mexico	Flu

### K-Anonymity

To protect data from a linking attack, Samarati and Sweeny proposed k-anonymity [7]. This method generalizes or suppresses the QI attributes so that each record is indistinguishable from at least k-1 other records within the dataset. The larger the value of k, the greater the implied privacy, since no individual can be identified with probability exceeding 1/k through linking attacks alone. For example, Table 1 is the original data table, and Table 2 is an anonymized version of it that satisfies 3-anonymity. 3-anonymity means that at least three instances are identical with respect to QI. We can find that Q-blocks are made by generalizing QI attributes to satisfy 3-anonymity.

**Table 2:** An example of a 3-anonymous data table after generalization.

Index	Quasi-identifier (QI)		Sensitive
	Work	Country	Disease
1	Private	North	Heart Disease
5	Private	North	Heart Disease
8	Private	North	Heart Disease
2	Government	South	Cancer
3	Government	South	Cancer
9	Government	South	Flu
4	Workclass	North	Flu
6	Workclass	North	Heart Disease
7	Workclass	North	Flu

Therefore, k-anonymity is defined as: Let *D* denote the original data table and *D* ^*^ denote a release candidate of *D* produced by the generalization. Given a set of QI attributes *Q*_*1*_,…,*Q*_*d*_ , release candidate *D* ^*^ is said to be k-anonymous with respect to *Q*_*1*_,…,*Q*_*d*_ if each unique tuple in the projection of *D* ^*^ on *Q*_*1*_,…,*Q*_*d*_ occurs at least *k* times.

### L-Diversity

While k-anonymity protects against linking attacks, it does not provide sufficient protection for sensitive attributes. This has been recognized by previous studies. The following two attacks are presented to show a homogeneity attack and a background knowledge attack [11].

**Homogeneity Attack**

In an anonymized table, if a Q-block exists in which all tuples share the same value of sensitive attributes, it will be exposed to a homogeneity attack because an adversary can easily infer an individual’s sensitive value by linking an external table.

**Background Knowledge Attack**

An adversary can infer individuals’ sensitive information from an anonymity table using his/her background knowledge. In order to guarantee privacy against such adversaries, Machanavajjhala et al proposed the *l*-diversity principle.

Machanavajjhala et al indicate that *l*-diversity can resist homogeneity and background knowledge attacks [11]. *l*-diversity is defined as: A Q-block is said to have *l*-diversity if it contains at least *l* “well-represented” values for sensitive attribute. A table is said to have *l*-diversity if every Q-block has *l*-diversity. Table 3 is an example of a 3-diverse data table. Machanavajjhala et al provide a number of interpretations of the term “well-represented.”

**Table 3:** An example of a 3-diverse data table.

Index	Quasi-identifier (QI)		Sensitive
	Work	Country	Disease
1	Workclass	America	Heart Disease
3	Workclass	America	Cancer
7	Workclass	America	Flu
2	Workclass	America	Cancer
8	Workclass	America	Heart Disease
9	Workclass	America	Flu
4	Workclass	North	Flu
5	Workclass	North	Heart Disease
6	Workclass	North	Heart Disease

**Distinct** ***l-*** **diversity**

**Figure 1:**
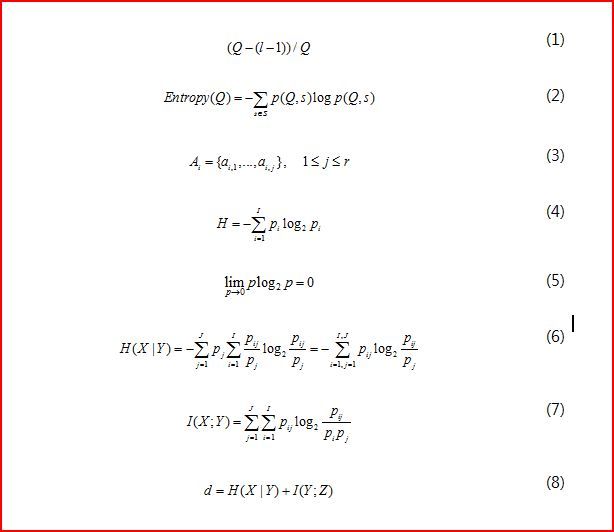
Equations (1) to (8).

The simplest understanding of “well represented” would be to ensure that there are at least *l* distinct values for the sensitive attribute in each Q-block. It can guarantee that the sensitive value is predicted correctly by the adversary as equation (1), where *Q* is the number of rows in the Q-block (see Equation (1) in Figure 1). However, distinct *l*-diversity cannot provide a stronger privacy guarantee because when Q-block may have one value that appears much more frequently than other values, an adversary would be able to predict that an entity in the Q-block is most likely to have that value. This motivated the development of the following two stronger notions of *l*-diversity.

**Entropy** ***l*** **-diversity**

When *s* is the domain of the sensitive attribute, and *p*(*Q*, *s*) is the fraction of instances in *Q* (Q-block) that have sensitive value *s*, Equation (2) represents the entropy of a Q-block (see Figure 1). A table is said to have entropy *l*-diversity if all Q-blocks satisfy “*Entropy*(*Q*)≥log *l*”. Entropy *l*-diversity is stronger than distinct *l*-diversity. In order to have entropy *l*-diversity for each Q-block, the entropy of the entire table must be at least log *l*. Sometimes this may be too restrictive, as the entropy of the entire table may be low if a few values are very common. This leads to the following less conservative notion of *l*-diversity.

**Recursive (** ***c*** **,** ***l*** **)-diversity**

Recursive (*c,l*)-diversity ensures that the most frequent value does not appear too frequently, and the less frequent values do not appear too rarely. Let *m* be the number of values in a Q-block and *r*_*i*_(1≤*i*≤*m*) be the number of times that the *i*_*th*_ most frequent sensitive value appears in a Q-block. Then Q-block is said to have recursive (*c,l*)-diversity if *r*_1_<(*r*_*l*_+*r*_*l*__+1_+…+*r*_*m*_)*.* A table is said to have recursive (*c,l*)-diversity if all of its Q-blocks have recursive (*c,l*)-diversity.

### Limitations of Recent Studies

While the *l*-diversity principle represents an important step beyond k-anonymity for protecting against attribute disclosure, it has several shortcomings. We have already explained that the Q-block is made by generalizing database entries. Generalization of QI attributes leads to an information loss, so minimizing information loss is a very important issue. However, most recent *l*-diversity studies focus only on diversifying sensitive attribute without accounting for information loss of QI attributes. It means that they consider k-anonymity and *l*-diversity independently.

Li et al proposed the *t*-closeness method, which protects against sensitive attributes disclosure by defining semantic instance among sensitive attributes [12]. This approach requires distance between the distribution of the sensitive attribute in the group and the distribution of the attribute in the whole dataset to be no more than a threshold *t*. However *t*-closeness would greatly damage the data utility when *t* is small because enforcing *t*-closeness destroys the correlations between quasi-identifier attributes and sensitive attributes [17,18]. Other recent studies proposed privacy protection methods, which handle k-anonymity and *l*-diversity [19,20]. These studies proposed an improved algorithm to reduce the complexity or efficient implementation. However, the methods they have proposed improve only the individual performance of k-anonymity and *l*-diversity.

Data utility and sensitivity disclosure have to be considered for actual EHR data release. Therefore, research that covers both characteristics of k-anonymity and *l*-diversity is necessary. As such, we have developed a method that considers both algorithms (k-anonymity and *l*-diversity).

## Methods

We have indicated some of the limitations of k-anonymity and *l*-diversity in the previous section. In this paper, we propose a method to make a Q-block that minimizes information loss while achieving diversity of sensitive attributes. For this, we use two measurements: conditional entropy and mutual information. These two measurements are based on entropy characteristics. The use of conditional entropy to obtain minimum information loss has already been studied [13,21]. However, this method cannot guarantee the diversity of sensitive attributes. Therefore, we use conditional entropy as well as mutual information to calculate the distance between instances in order to offer an appropriate Q-block. Mutual information is a quantity that measures a relationship between generalized and sensitive attributes. Therefore, choosing a set that has a small value for mutual information can achieve the required diversity of sensitive attributes.

To calculate the conditional entropy and mutual information, we assume that a dataset holds information on an individual from a population *D* ={*D*_*1*_,…,*D*_*n*_}. Each individual consists of a collection of QI attributes and sensitive attributes. In this paper, when *i* is the index of attribute, *r* is the total number of attributes, and *j* is the number of possible values, we will treat both attributes and define these as Equation (3) (see Figure 1). If *A*_*i*_ is *work* class, then *A*_*i*_ ={*Private*, *Self-emp-not-inc*,…, *Never-Worked*}.

**Figure 2:**
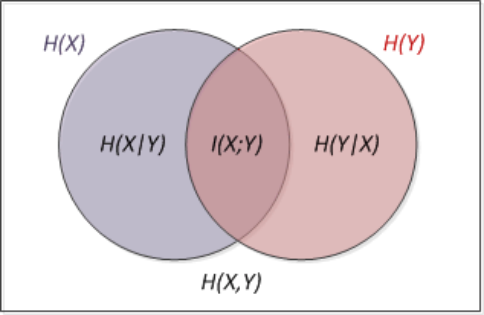
Individual conditional entropies and mutual information for a pair of correlated subsystems.

Figure 2 shows the individual conditional entropies and mutual information. Entropy *H* equals the negative of the sum of category probabilities times the logarithms of category probabilities, where *i* is a particular value of attribute. See Equations (4) and (5) in Figure 1.

The value *H* lies between 0 and log_2_ *I*. It is zero only when the value of one of the *p*_*i*_s is one and all the others zero. Conditional entropy quantifies the remaining entropy of a random variable *X*, given that the value of another random variable *Y* is known [22,23]. Where *p*_*i j*_ is joint probability distribution, conditional entropy is referred to as the entropy of *X* conditional on *Y* (see Equation (6) in Figure 1).

To make a Q-block that satisfies 3-anonymity, we have to generalize the set that contains at least three instances. We chose these instances to calculate the distance between all possible pairs of instances. A small distance value means that they are close to each other. If attribute *X* in the original database is generalized into *Y*, then *H(X|Y)* indicates the information loss by generalization. In order to minimize information loss, we use conditional entropy to calculate the distance.

For example, suppose we generalize Table 1. Assume that the Q-block is built with respect to the first instance. As a first step, we calculate the distance between the first instance and others. Second, we find instances that are close to the first instance using the results of distance. Table 4 shows an example of generalizing between first instance and second instance. In this case, *private* and *state-gov* of the *Work* attribute are generalized into *Workclass*. We calculate the conditional entropy between the original *Work* attribute and generalized *Work* attribute. Next, we perform the same process to the *Country* attribute. The sum of two conditional entropy values is the distance between the first instance and second instance and is expressed as *d*_*1,2*_ *.* We calculate distances *d*_*1,2*_ ~ *d*_*1,9*_ *,* which are all possible pairs of instances and then choose two instances that have minimum values. Generalizing these selected instances can make a Q-block with minimum information loss.

**Table 4:** Data table showing generalized QI attributes and sensitive attributes for first instance and second instance to explain conditional entropy and mutual information.

Index	Original quasi-identifier		Generalized quasi-identifier		Sensitive
	Work	Country	Work	Country	Disease
1	Private	USA	Workclass	America	Heart Disease
2	State-gov	Mexico	Workclass	America	Cancer
3	Local-gov	Brazil	Local-gov	Brazil	Cancer
4	Federal-gov	USA	Federal-gov	USA	Flu
5	Private	Canada	Private	Canada	Heart Disease
6	Self-emp-not-inc	Canada	Self-emp-not-inc	Canada	Heart Disease
7	Self-emp-inc	USA	Self-emp-inc	USA	Flu
8	Private	USA	Private	USA	Heart Disease
9	State-gov	Mexico	State-gov	Mexico	Flu

However, this method using only conditional entropy cannot prevent homogeneity attacks or background knowledge attacks. Therefore, the proposed method uses mutual information in addition to conditional entropy to achieve diversity of sensitive attributes. Mutual information is a general measure of dependence between two random variables [22,23]. It can be defined as Equation (7) (see Figure 1).

Mutual information is a useful concept for measuring the amount of information shared between a generalized database and sensitive attributes [24,25]. A low value of mutual information indicates that the generalized database and sensitive attributes are almost independent. In order to achieve diversity of a sensitive attribute, we use mutual information to calculate the distance.

We showed an example of calculating information loss by generalization between first instance and second instance using conditional entropy. Table 4 shows an example that calculates mutual information between generalized QI attributes and the sensitive attribute. We calculated the joint probability distribution of QI attributes and the probability distribution of sensitive attributes to achieve mutual information. Mutual information can measure the similarity of the probability distribution between QI attributes and the sensitive attribute. When the first instance and second instance are generalized, their QI values are changed to the same value. In this case, the mutual information {*Heart Disease, Heart Disease*} of the sensitive attribute is larger than {*Heart Disease, Cancer*}. Therefore, to achieve diversity of the sensitive attribute, we made a Q-block that has lower mutual information between the generalized database and the sensitive attribute.

**Figure 3:**
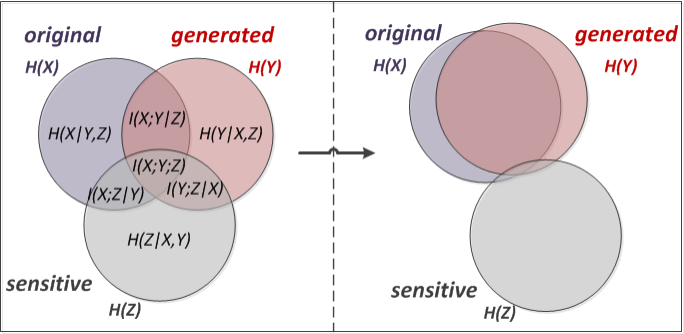
Simplified concept of the proposed method.

We can now explain the concept in a more detailed manner. Figure 3 shows the set that minimizes conditional entropy between the original database and the generalized database and mutual information between the generalized database and the sensitive attribute. The distance function, defined as Equation (8) (see Figure 1), measures the information loss and diversity. We chose instances that have the smallest value of Equation (8) to make appropriate Q-blocks. The total information loss can be calculated by summing up the loss of all Q-blocks.

Algorithm 1 (see Appendix 1) shows the procedure of calculating distance. Let *S* = {*s*_*i*_}_1≤__*i*__≤__*N*_ be the set of instances, where *N* is number of instance. *s*_*ik*_ is the *k* ^th^ attribute value of *i* ^th^ instance of *S*. When *i* ^th^ and *j* ^th^ instances are generalized, the total conditional entropy is the addition of each attributes conditional entropy value. Next the mutual information between QI and sensitivity attribute is calculated. During this step, the combinational values of QI are considered as s single value for mutual information calculation. Total distance between two instances will be the sum of mutual information and total conditional entropy.

We have used a simple clustering method to construct a dataset that satisfies the k-anonymization (see Algorithm 2 in Appendix 1). First randomly select an instance as a seed, and then subsequently select and add k-1 records to build the Q-block. The distance is calculated based on Algorithm 1.

## Results

We used the adult database from the UCI Machine Learning Repository in our experiments. This database contains census data and has become a commonly used benchmark for k-anonymity. This dataset consists of 15 attributes and 30,162 samples (patients), and we used 9 attributes where numeric attributes are not included. For the adult database, we used Occupation as the sensitive attribute and other attributes as the QI attributes. All methods are implemented in Java and run on a PC with Quad 2.4GHz processor and 4GB RAM under the Windows 7 operating system.

**Figure 4:**
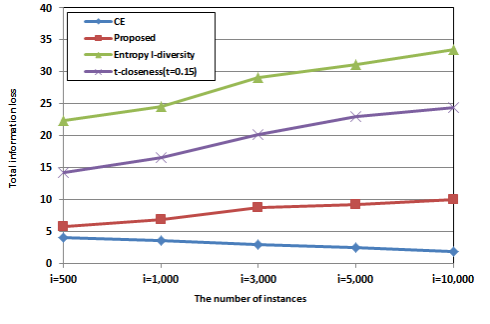
Comparison of total information loss with respect to the number of instances.

Figure 4 presents the performance of total information loss when the Q-block size is 3. The x-axis is the number of instances and the y-axis is the total information loss. We compared the proposed method with k-anonymity using conditional entropy (CE), entropy *l*-diversity, and *t*-closeness(*t* = 0.15). Total information loss of CE is decreased, associated with the number of instances. The large number of instances leads to a stochastic reduction in the average value of *p*_*i j*_ in Equation (6) (see Figure 1). In addition, more of the same attribute values can be obtained by increasing the number of instances, in which case conditional entropy is zero, so total information loss is not increased. However, total information loss of entropy *l*-diversity, *t-*closeness, and the proposed method is increased in response to the larger number of instances. Even though *p*_*i j*_ is reduced with a large number of instances, entropy *l*-diversity, in particular, generalizes targets in proportion to the number of instances, so information loss is increased.

The proposed method shows some degradation of information loss when compared with CE. Even though the proposed method considers the information loss, it cannot surpass CE because the proposed method uses conditional entropy but also mutual information to make the Q-block. This means that the proposed method considers information loss to a lesser extent than does CE. However, the proposed method is more than four times better than entropy *l*-diversity. It also shows better (nearly three times better) performance compared to the *t*-closeness method (*t* = 0.15).

**Figure 5:**
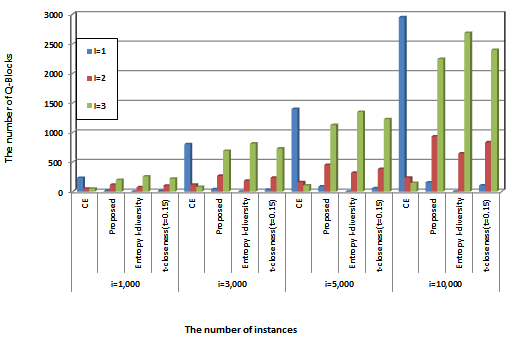
Comparison of the number of Q-blocks, which are l=1 (homogeneity attack), l=2 (background knowledge attack), and l=3 (safe), to measure the diversity (the size of Q-block is set to 3).

Figure 5 presents the number of Q-blocks for “*l* = 1, 2, 3”. The x-axis is the number of instances, and the y-axis is the number of Q-blocks. We compare the proposed method with CE, entropy *l*-diversity, and *t*-closeness (*t* = 0.15). We have already explained that k-anonymity is susceptible to homogeneity attacks and background knowledge attacks. Assuming that the size of the Q-block is 3, a homogeneity attack will occur when *l* equals 1, and a background knowledge attack will occur when *l* equals 2. Therefore, we can confirm that reducing the number of Q-blocks for “*l* = 1, 2” represents a higher diversity of sensitive attributes. In Figure 5, the proposed method reduces the number of Q-blocks for “*l* = 1, 2” when compared with CE. We also found that the proposed method showed similar performance with *t*-closeness. However, the proposed method is somewhat inferior to entropy *l*-diversity in performance. From these results, we confirmed that the proposed method can reduce information loss while retaining diversity of sensitive attributes.

**Figure 6:**
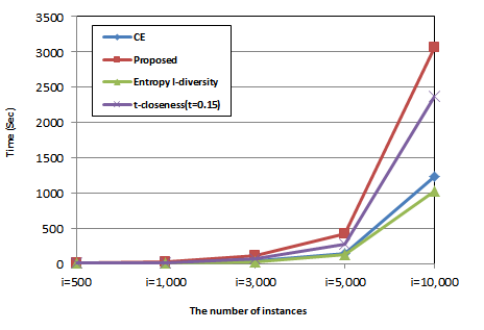
Comparison of execution time with respect to the number of instances.

Figure 6 presents the execution time and compares the proposed method with CE, entropy *l*-diversity, and *t*-closeness (*t* = 0.15). The x-axis is the number of instances, and the y-axis is the execution time. We found that CE and *l*-diversity give almost the same performance, whereas the proposed method and *t*-closeness are slower than the other two methods (ie, CE and entropy *l*-diversity). The reason is that the proposed method calculates mutual information, and calculating the joint probability distribution is quite complex. This shows similar complexity level with KL-divergence calculated by *t*-closeness [12]. Therefore, the proposed method and *t*-closeness share a similar performance. Although our method is slower than others, the overhead is still acceptable in most cases considering its better performance with respect to the information loss and diversity.

## Discussion

### Limitations

We have used mutual information to achieve diversity of sensitive attributes. From the experimental results, we confirmed that the proposed method can reduce the probability of homogeneity and background knowledge attacks. However, there is still room for improvement. The proposed method can substantially increase the diversity, while metrics for calculating the increment of diversity is left for further study. Also, similarity attacks must still be considered. When the sensitive attribute values in a Q-block are distinct but semantically similar, an adversary can learn privacy information. We need to carry out further work to address these problems, and then we will be able to provide even better improvements in privacy protection in EHR applications.

### Conclusions

This paper proposes a new privacy protection method that uses conditional entropy and mutual information. This method not only minimizes information loss but also achieves diversity of the sensitive attribute. This leads to increased data usability and prevents homogeneity attacks. This method was experimentally verified using an adult database from the UCI Machine Learning Repository. We compared the proposed method with previous *l*-diversity methods (ie, entropy *l*-diversity and *t*-closeness) to show that our method enables a reduction in information loss. It also can guarantee diversity of sensitive attributes when compared with CE. The method is viable and should be of interest for further utilization of privacy protection in various EHR data applications.

## Multimedia Appendix 1

Algorithms 1 and 2.
