# Supplementary material for: An Approach to Reducing Information Loss and Achieving Diversity of Sensitive Attributes in k-anonymity Methods
Source: Interact J Med Res. 2012 Nov 13;1(2):e14. doi: 10.2196/ijmr.2140 (PMC3626125; doi:10.2196/ijmr.2140)
Supplement: Supplementary file 1 [file ijmr_v1i2e14_app1.pdf]

Multimedia Appendix 1. Algorithms 1 and 2.

Algorithm 1. Calculating distance.

Input: a set of instance *S*, target instances s_i and s_j

Output: distance between s_i and s_j

let *r* = the number of attributes;

for *k* = 1 to *r*;

if (s_{ik} == s_{jk});

ce = 0;

else;

s_{ik}, s_{jk} = find generalization value (s_{ik}, s_{jk});

ce = calculate conditional entropy (s_{ik}, s_{jk}, S_k);

total_ce += ce;

mi = caclulate mutual information (s_i, s_j, *S*);

distance = total_ce + mi;

return distance;

Algorithm 2. k-anonymization

Input: a set of instance *S* and a threshold value k

Output: a set of Q-blocks each of which contains at least k

let *p* = number of instances;

for *i* = 1 to *p*;

r = a randomly picked instance from *S*;

for *j* = 1 to *k*-1;

c = find closest instance from r using algorithm 1;

```
Q-Block = generalization c and r;
result = result ∪ Q-Block;
S = S – c – r;
return result;
```
